# Supplementary material for: Risk factors for cerebral complications in patients with pulmonary arteriovenous malformations: A multicenter retrospective cohort study
Source: PLoS One. 2022 Dec 1;17(12):e0278610. doi: 10.1371/journal.pone.0278610 (PMC9714939; doi:10.1371/journal.pone.0278610)
Supplement: S1 Table — (DOCX) [file pone.0278610.s002.docx]

**S1 Table: Characteristics of patients with cerebral complications**

| No | Age | Sex | Incidental detection | HTN | DM | FAD  (mm) | Venous sac size (mm) | Types of PAVM | Location | Cerebral complications  before diagnosis PAVM | Cerebral complications with diagnosis PAVM | Cerebral  complications after diagnosis PAVM | mRS | Initial  treatment | Delayed treatment |
| --- | --- | --- | --- | --- | --- | --- | --- | --- | --- | --- | --- | --- | --- | --- | --- |
| 1 | 65 | F | No | No | No | 1.8 | 7.8 | Single | RLL | No | No | Stroke | 5 | No | No |
| 2 | 44 | F | No | No | No | 4.3 | 17.3 | Single | RLL | No | Stroke | No | 4 | Embolotherapy | No |
| 3 | 60 | F | Yes | No | No | 3.6 | 11.8 | Multiple | LUL, LLL | Stroke | No | No | 1 | Embolotherapy | No |
| 4 | 77 | F | Yes | No | No | 3.8 | 19.1 | Single | LUL | No | No | Stroke | 1 | No | No |
| 5 | 78 | F | No | No | No | 4.9 | 23.3 | Single | RML | No | Stroke | No | 2 | No | No |
| 6 | 91 | F | Yes | Yes | No | 4.4 | 7.5 | Single | RLL | Stroke | No | No | 1 | No | No |
| 7 | 53 | F | No | Yes | No | 4.4 | 13.2 | Single | LLL | No | Stroke | Stroke | 3 | No | Embolotherapy |
| 8 | 80 | F | No | Yes | Yes | 6.6 | 22.4 | Single | RML | Stroke | No | No | 4 | Embolotherapy | No |
| 9 | 80 | F | No | Yes | No | 4.7 | 8.6 | Single | LLL | No | Cerebral abscess | Stroke | 1 | No | Embolotherapy |
| 10 | 61 | F | Yes | No | No | 5.4 | 11.8 | Multiple | RLL, LLL | Stroke | No | No | 1 | No | No |
| 11 | 56 | F | No | No | Yes | 4.2 | 7.6 | Multiple | LUL, LLL | Stroke | No | No | 0 | No | No |
| 12 | 62 | F | No | No | No | 5.8 | 8.7 | Single | RUL | No | Stroke | No | 1 | Embolotherapy | No |
| 13 | 53 | F | No | Yes | No | 4.1 | 11.2 | Single | LUL | Stroke | No | No | 1 | Embolotherapy | No |

No, number; HTN, hypertension; DM, diabetes mellitus; FAD, feeding artery diameter; PAVM, pulmonary arteriovenous malformation; mRS, modified Rankin Scale; F, female; RLL, right lower lobe; LUL, left upper lobe; LLL, left lower lobe; RML, right middle lobe; RUL, right upper lobe
