# Supplementary material for: Risk factors for cerebral complications in patients with pulmonary arteriovenous malformations: A multicenter retrospective cohort study
Source: PLoS One. 2022 Dec 1;17(12):e0278610. doi: 10.1371/journal.pone.0278610 (PMC9714939; doi:10.1371/journal.pone.0278610)
Supplement: Attachment — Submitted filename: Response to Reviewers.docx [file pone.0278610.s003.docx]

**PONE-D-22-08058**

**Risk factors for cerebral complications in patients with pulmonary arteriovenous malformations: a multicenter retrospective cohort study**

PLOS ONE

**Responses to reviewers**

**Reviewer #1:** The authors seek to address an important issue in PAVM pathophysiology—the risk of developing cerebral complications—especially when the primary pathology is asymptomatic and incidental. The authors acknowledge their biggest limitation which is the small sample size of their population. Unfortunately, the small sample size underpowers their statistical analysis as can be seen in the wide range of their confidence interval. As such, I am concerned that their claims, especially with their subgroup analysis, is overstated. Nonetheless, this serves as a starting point for future studies. I would recommend that the authors strengthen their focus on the types of neurologic complications and highlight patient outcomes before this paper is suitable for acceptance.

A. Thank you for your precise and kind comments. This study only included a small number of participants because PAVM is a rare disease. We believe this study can serve as a starting point for diagnosing and treating of PAVM. We tried to strengthened the types and outcomes of neurologic complications, as you pointed out.

**Q1. Study Design: I think the authors need to provide more details on how the patients were selected. Why were these patients with “incidental PAVMs” who are presumably asymptomatic scanned w a CT or pulmonary angiography? Would that introduce a population bias?**

A1. Thank you for your comment. We screened patients with PAVM confirmed by diagnostic code, chest computed tomography (CT) findings, or pulmonary angiography findings during the study period. A total of 70 individuals were screened, of which 15 were excluded due to the following reasons: CT or angiography did not reveal PAVM (n = 11), and diagnosis of pulmonary sequestration (n = 3) or pulmonary venous anomaly (n = 1). A total of 55 patients with PAVM were enrolled in this study.

We added this information to method and results sections. See line 57-59 on page 4 and line 109-112 on page 7.

**Q2. Results line 110-111: Since the authors want to describe the risk of cerebral complications w PAVMs, I think it is important for them to describe the types of cerebral complications they had in their populations. They only provide a binary description of symptomatic and incidentally detected.**

A2. Thank you for your valuable comment. We added the types of complications in the results section, as your recommendation. See line 120-123 on page 7.

**Q3. Results line 115-116: Would recommend describing the complications that occurred in the patients who did not undergo treatment (were they considered more severe? Any pattern?)**

A3. Thank you for your valuable comments. Cerebral complication in all the four patients included strokes, and one of them showed severe functional impairments, with a mRS score of 5. The other three, however, only showed mild impairments with mRS scores of 1¬3. We added this information to method and results sections. See line 126-132 on page 7-8.

**Q4. Results line 115-116: The authors state a total of 13 pts had cerebral complications and of them, four patients did not undergo treatment. Subsequently, in line 117-118, they state that none of the patients who underwent treatment for PAVMs had cerebral complications. Please provide clarification or additional details on the remaining 9 patients with complications.**

A4. Thank you for your valuable comments. In this study, of the 13 patients with cerebral complications, 12 had stroke and one had a cerebral abscess; moreover, six developed cerebral complications before PAVM diagnosis, and five developed them concurrently with PAVM diagnosis. Among patients with untreated PAVM, four patients developed cerebral complications after diagnosis of PAVM, and two of those had previous cerebral complications. We modified the results section as your recommendation. See line 120-123 on page 7.

**Q5. Would recommend providing a table detailing the demographics of the patients with cerebral complications and their outcomes**

A5. Thank you for your comments. We added a S1 Table for 13 patients with cerebral complications. See S1 Table.

**Q6. Table 2 reports several risk factors based on characteristics of PAVMs (feeding artery diameter, types of AVM) but does not report size or location which may be useful to include.**

A6. Thank you for your valuable comments. PAVM locations and size of venous sac have been added to tables 1 and 2. However, both variables were not risk factors for development of cerebral complications in the univariate logistic regression analysis. See table 1 and 2 on page 8 and 11, respectively.

**Q7. Figure 2: Is a Kaplan Meier survival curve the best choice in trying to highlight the morbidity of cerebral complications especially since there was no survival difference? It may be more meaningful to look at functional scales such as mRS or GOS.**

A7. Thank you for your comments. Figure 2 shows a Kaplan-Meier curve of time without cerebral complications following treatment of pulmonary arteriovenous malformation. Although the untreated group developed more cerebral complications during the follow-up period, there was no significant difference between the two groups (log-rank P = 0.088). Due to the small number of patients enrolled, we did not consider the difference to be statistically significant.

Based on your opinion, we measured the modified Rankin Scale (mRS) of patients with cerebral complications after detection of PAVM. In four patients, cerebral complications developed after PAVM detection, and the mRS varied from 1, 1, 3, and 5, respectively, therefore the number of patients was insufficient for a statistical analysis. These findings have been added as limitation part. See line 260-262 page on 17.

**Q8. Line 191: Missing the word echo for TTCE**

A8. I appreciate your kind words. The error has been corrected.

**Reviewer #2**

**Q1. In the conclusions section, there is no mention of the possibility that both hypertension and diabetes may also be a contributing factor given that one or other were present in all patients with cerebral complications with pAVM. Perhaps comment as to why it is not relevant or consider that in fact may well be.**

A1. Thank you for your valuable comments. Diabetes and hypertension are well-known risk factors for stroke. In this study, we investigated whether diabetes and hypertension were risk factors for stroke among PAVM patients. Table 2 of this study shows risk factors associated with cerebral complications. Hypertension and diabetes were not statistically significant in univariate logistic regression analysis in this study. However, there were a few patients with hypertension or diabetes in this study cohort (11 with hypertension and three with diabetes). Therefore, multicenter studies that allow the inclusion of a larger number of patients with PAVMs are needed. This was added to the limitation. See line 263-267 on page 17.
