# Supplementary material for: Risk factors for cerebral complications in patients with pulmonary arteriovenous malformations: A multicenter retrospective cohort study
Source: PLoS One. 2022 Dec 1;17(12):e0278610. doi: 10.1371/journal.pone.0278610 (PMC9714939; doi:10.1371/journal.pone.0278610)
Supplement: Attachment — Submitted filename: Response to Editor.docx [file pone.0278610.s004.docx]

**PONE-D-22-08058**

**Risk factors for cerebral complications in patients with pulmonary arteriovenous malformations: a multicenter retrospective cohort study**

PLOS ONE

**Responses to editor**

**Q1 - The reviewer asks the medical rationale for workup and diagnosis of patients without symptoms that led to PAVM. In lines 57-59, you provide information on how these patients were identified from the medical record system, but not why they were evaluated with CT or angiography. "More than half of the enrolled patients were diagnosed with PAVMs incidentally without symptoms."**

**A1. Thank you for your precise and kind comments.**

A definition of incidentally detected PAVM has been added to the method section as "Patients with incidentally detected PAVM were those whose PAVM was detected during a health screening, preoperative evaluation, or evaluation of other diseases, and presented asymptomatically. ". [See line 84-57 on page 5]

Thirty patients were incidentally detected PAVM due to health screening (n = 18), preoperative evaluation (n = 6), and evaluation of other diseases (n = 6) such as hepatocellular carcinoma, Sjogren syndrome, External MALToma, AOSD, back pain, or hoarseness. These findings have also been added to the results section [See line 127-129 on page 7]

**Q2-1- The reviewer asks for more specifics of the type of cerebral complication, and you now explain stroke vs. abscess. More detail is likely appropriate again, from the perspective of this reviewer as a neuroscience expert. For example, presumably these are ischemic strokes, although hemorrhagic stroke may also be possible.**

**A2-1. Thank you for your comments**. There were 11 patients with cerebral ischemia and one patient with cerebral hemorrhage. We change the manuscript as “A total of thirteen patients (23.6%) had cerebral complications, including cerebral ischemia (n = 11), cerebral hemorrhage (n = 1), and cerebral abscess (n = 1).”. [See line 129-130 on page 7]

**Q2-2. Furthermore, in discussing cerebral complications at the time of diagnosis (13 patients), the authors now report 9 symptomatic vs 4 asymptomatic, and 12 strokes vs 1 abscess, but describe 6 before diagnosis and 5 at the time of diagnosis (adding up to 11, not 13).**

**A2-2. Thank you for your comments.**

It has caused confusion to use words such as "incidentally detected", "symptomatic", and "asymptomatic.". The term "asymptomatic patients" meant the same thing as incidentally detected patients.

We added the definition of incidentally detected PAVM on the method section. [See page 5, line 84-87] In addition, sentences with unclear meanings were removed. [See page 7, line 131-133], and see page 8, line 144-145 of revised manuscript with track change file]

The discussion section was also edited. [See line 213-217 on page 15]

**Q2-3. The characterization of complications before pAVM diagnosis merits further clarification in the methods.**

**A2-3. Thank you for your comment.** We added the clarification of cerebral complications to the method section as “The cerebral complications associated with PAVM were divided into cerebral ischemia, hemorrhage, and abscess.”. [See line 74-75 on page 4]

**Q2-4. Are these prior strokes based on brain imaging (not described in methods)?**

**A2-4. Thank you for your comment**. In order to determine whether there had been any brain complications before PAVM diagnosis, we reviewed the patient's medical records. However, brain CT or MRI was used to diagnose cerebral complications associated with PAVM at the time or after detected PAVM. We modified the method section as “A brain CT or MRI was used to diagnose cerebral complications associated with PAVM at the time of initial PAVM diagnosis or after PAVM was detected.” [See line 76-78 on page 5]

**Q2-5. The reviewer asks for a more direct description of:**

**pAVM - presenting asymptomatically**

**pAVM - presenting with symptoms unrelated to the brain**

**pAVM - presenting with neurologic symptoms**

**pAVM untreated patients - risk of subsequent neurologic symptoms**

**pAVM treated patients - risk of subsequent neurologic symptoms**

**Part of the problem appears to be semantic. The authors use the same term, cerebral complication, to describe strokes before diagnosis, strokes which were the presenting symptom leading to diagnosis, and strokes which occur after diagnosis. These are then used in one large paragraph. A flow chart of the events or a timeline plot of events may provide better clarity. For example Figure 1 in https://www.ncbi.nlm.nih.gov/pmc/articles/PMC7606530/**

**A2-5. Thank you for your valuable comments.**

There was some confusion in the terminology we used, as you pointed out. In the method section, "PAVM presenting asymptomatically" is defined as “Patients with incidentally detected PAVM were those whose PAVM was detected during a health screening, preoperative evaluation, or evaluation of other diseases, and presented asymptomatically.”. [See line 85-57 on page 5]

In our study, we did not distinguish between PAVMs with symptoms unrelated to the brain and PAVMs with neurologic symptoms.

We clarified the cerebral complications associated with PAVM. We evaluated the risk factors for cerebral complications, including cerebral hemorrhage and ischemia, at the time of PAVM diagnosis (Table 2). Figure 2 shows the Kaplan–Meier curve for the development of new cerebral complications in the treated and untreated groups after PAVM diagnosis. We investigated the risk factors associated with new cerebral complications in 33 patients who did not receive treatment after their PAVM diagnosis (Table 3). Figure 3 shows the Kaplan–Meier curve for the development of new cerebral complications in the ≥ 65 years and < 65 years age groups of patients who did not undergo treatment after a PAVM diagnosis. The results section has been edited. [See line 159-160 on page 10, line 171-172 on page 12, line 177-183 on page 12, line 187-188 on page 13, line 191-193 on page 13, line 199-200 on page 14]

Despite your recommendation, we were not able to create a timeline plot because the timing of past cerebral complications was unclear (retrospective study).

**Q3. - S1 provides no legend for interpretation of the coded data or column names. For example, column D "Detect_reasons" has codes 1-9. The reader would benefit from seeing the 9 reasons that pAVM is presenting for evaluation. Table 1 only has 8 'causes of detection'**

**A3. Thank you for your comments.** We modified detect reasons of S1 files. The reason 1 and 2 are belongs to incidentally categories (1: health screening, 2: preoperative evaluation or other disease evaluations).
