# Supplementary material for: Risk factors for cerebral complications in patients with pulmonary arteriovenous malformations: A multicenter retrospective cohort study
Source: PLoS One. 2022 Dec 1;17(12):e0278610. doi: 10.1371/journal.pone.0278610 (PMC9714939; doi:10.1371/journal.pone.0278610)
Supplement: Attachment — Submitted filename: Response to editor and reviewer.docx [file pone.0278610.s005.docx]

**PONE-D-22-08058R2**

**Risk factors for cerebral complications in patients with pulmonary arteriovenous malformations: a multicenter retrospective cohort study**

**<Response to editor>**

Q1) Please review your reference list to ensure that it is complete and correct. If you have cited papers that have been retracted, please include the rationale for doing so in the manuscript text, or remove these references and replace them with relevant current references. Any changes to the reference list should be mentioned in the rebuttal letter that accompanies your revised manuscript. If you need to cite a retracted article, indicate the article’s retracted status in the References list and also include a citation and full reference for the retraction notice.

A1) Thank you for your comments. All references cited in this article have been thoroughly reviewed. We have also mentioned additional references below.

**Additional Editor Comments:**

Q2) Thank you for your revisions. Due to reviewer availability, additional reviewers were requested. They shared a similar concern regarding the possibility of comorbid risks for stroke in the older patient population. It would be appropriate to explicitly state in the limitations that the observed cerebral complications cannot be definitively linked to the PAVM, although this is assumed in the analysis.

A2) Thank you for your comments. We understand the concerns of reviewers and editors. We have added these concerns to the limitation section with reference as your suggestion. (See line 278-280 on page 18.)

Q3) For Table 3 the multivariate analysis section and the related results text only applies the statistical test (multivariate logistic regression) to a single variable (Age) therefore, it is not different from the univariate logistic regression, and logically, reports the same OR, CI, and p-value. Since no other co-variates were included in the 'multivariate' analysis, it is misleading to present this as a multivariate analysis and to re-present the data in a separate section of the table.

A3) Thank you for your valuable comments. We mentioned follows in the statistical analysis part of the methods section: Subsequent multivariate logistic regression analyses included variables with P values < 0.1 in the univariate analysis using a backward method. We changed the variable to be selected in univariate analysis from P <0.1 to P <0.2 in the same way as in Cox-regression analysis. As a result, age >65 and hypertension were selected in the univariate analysis. Table 3 shows the results of multivariate analysis with age >65 and hypertension. Additionally, diabetes was also selected in univariate analysis in Table 2. After multivariate analysis, the results are presented in table 2. (See line 34 on page 2, 110 on page 6, line 162-164 on page 10-11, table 2, line 185 on page 13, and table 3)

* Reference 21 has been added as a recent article. (See line 217-219 on page 15, and line 255 on page 17)

**<Response to Reviewer #3>**

Q1) Reviewer #3: Cerebral and hemorrhagic complications associated with PAVMs are often fatal. This study retrospectively evaluated the risk factors for cerebral complications in patients with PAVMs. The included cerebral complications complications were stroke (12 cases) and brain abscess (one case ). The study was retrospective, and it is doubtful whether stroke is caused by PAVM in elderly patients.

A1) Thank you for your valuable comments. In this study, we understand the concerns of older patients. The risk of stroke in elderly patients is well known. Further studies are needed to determine whether strokes are more common in elderly patients with pulmoanry AVMs. We have added these concerns to the limitation section. (See line 278-280 on page 18.)
